# Current fertility desire and its associated factors among currently married eligible couples in urban and rural area of Puducherry, south India

**DOI:** 10.4314/ahs.v21i3.50

**Published:** 2021-09

**Authors:** Ganesh Kumar Saya, Kariyarath Cheriyath Premarajan, Gautam Roy, Sonali Sarkar, Sitanshu Sekhar Kar, Revathi Ulaganeethi, Jeby Jose Olickal

**Affiliations:** Department of Preventive and Social Medicine, Jawaharlal Institute of Postgraduate Medical Education and Research (JIPMER), Puducherry, India

**Keywords:** Fertility desire, eligible couples, India

## Abstract

**Background:**

There are paucity of studies on current fertility desire at community level.

**Objective:**

To assess current fertility desire and its associated factors among eligible couples of reproductive age group in Puducherry, India.

**Methods:**

A community-based cross-sectional study from 2016 to 2017 among 2228 currently married eligible couples assessed socio-demographic and fertility-related factors associated with fertility desire. Data were collected based on the National Family Health Survey questionnaire. Association of fertility desire was assessed by univariate and generalised linear regression analysis.

**Results:**

Out of 1979 respondents, current fertility desire within two years was 13.7% (95% CI, 12.3%–15.3%). Mean number of children (SD) currently living and preferred was 1.77(0.851) and 2.11 (0.528) respectively. After adjusting for confounders, the significant factors positively associated with fertility desire include woman's age of 18–24 (APR = 2.91), 25–29 years (APR=2.48), 30–34 (APR=2.47), 35–39(APR=2.06), high socioeconomic status (APR=2.02), those without child (APR=52.35) and those with one child (APR=35.60).

**Conclusion:**

The fertility desire is comparatively lesser than other areas. Those without or with a single child and high socioeconomic status group had comparatively more fertility desire.

## Introduction

Total Fertility Rate (TFR) has declined globally in recent years. The global TFR declined from 3.2 births per woman in the year 1990 to 2.5 births per women in the year 2019. In sub-Saharan Africa, TFR reduced from 6.3 to 4.6 during the same period 1. TFR has declined in South East Asian Countries also in two decades (from 1985–1990 to 2005–2010) except in Timor-Leste^2^. In India recent report showed that the TFR in rural areas has declined from 5.4 in the year 1971 to 2.4 in the year 2018 whereas the corresponding decline in urban areas has been from 4.1 to 1.7 during the same period^3^. Fertility desire is one of the important factors affecting TFR. Fertility desire can be predictor of contraceptive behaviour of women and fertility related outcomes 4–6. Magnitude of fertility desire has policy implications in formulating family planning strategies of countries 7.

Sustainable Development Goal targets 3.7 mentions that by the year 2030, ensure universal access to sexual and reproductive health-care services, including for family planning, information and education, and the integration of reproductive health into national strategies and programmes^8^. Therefore, assessment of fertility desire among eligible couples is an important parameter to be considered. The extent of fertility desire among eligible couples in the community will help concerned stakeholders to provide appropriate reproductive health care services according to their felt need.

Many studies on fertility desire were conducted among HIV infected eligible couples 9–18 and very few in the general population or community setting 19–20. These research findings mainly from African countries assessed fertility desire in future life and reported that majority (>50%) of the women have fertility desire. In India also, recent National Family Health Survey-4(2015–16) found that about 24% of women had fertility desire in their future life 21. Current fertility planning behavior of eligible couples is impotant to prevent unwanted pregnancies 22. It gives an idea about the proportion of women who wants childbearing currently or delay for more than two years and require spacing methods of contraception to prevent mistimed pregnancies 7. Fertility desire may change according to circumstances of personal or family characteristics. If conditions remain unfavourable, desires to postpone a birth persist and may lead to long birth intervals. Postponement desires could even translate into short birth intervals if conditions become favourable sooner than anticipated. Therefore, current fertility desire and the time taken for decision making to conceive among those with fertility desire is also an important aspect to be considered. Understanding current fertility desire patterns will enable health stakeholders to implement fertility-related services by meeting their desired number of children and improving maternal and child health services.

Fertility desire can be associated with number of factors related to individual, family or health care service availability characteristics. At the individual level, age, education, number of living children, number of living son or sex composition, history of abortion, age at marriage, marital life was the important associated factors 23. At family and community level, it depends on socioeconomic status, family type, religion, and service availability^23^. Although there are studies on factos affecting fertility desire in different settings, most studies have been conducted among HIV positive women. There is paucity of studies reported on current fertility desire and its associated factors from a general population except some national surveys^21^. Therefore, in this study, we assessed the prevalence and associated factors of current fertility desire among currently married women of reproductive age group in urban and rural Puducherry, India.

## Methods

### Ethics

This community-based cross-sectional analytical study was approved by Ethics and Scientific Committee of a tertiary care institution in Puducherry, coastal south India. Prior written permission was obtained from Deputy Director of Health Services, Government of Puducherry. Written informed consent was obtained from the study subjects.

### Study population, sample size estimation and sampling technique

This study is a part of a project which had assessed the contraception prevalence and factors associated with unmet need for family planning among currently married women of reproductive age group in selected rural and urban area of Puducherry, India. All the eligible couples were recruited by two-stage cluster sampling method.

Considering fertility desire as 63%^23^, with an absolute precision of 5%, and a design effect of 2, the calculated sample size for the study was 716. After adding a nonresponse rate of 20%, the minimum sample size required for the study was 895 each from urban and rural area. But we included all currently married women from the main study (N=2228) with 1114 each from urban and rural area.

**Figure 1:**
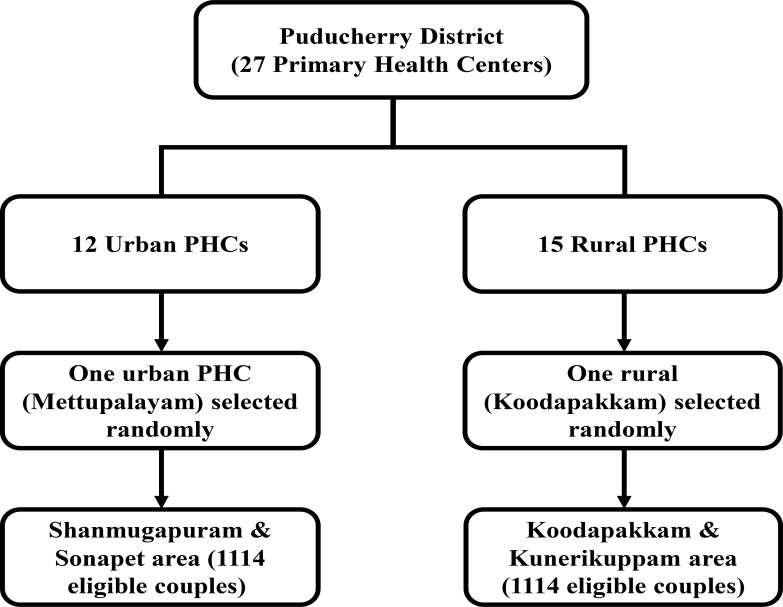
Sampling method for selection of participants

Sampling technique was summerised in Figure 1. There are 27 PHCs in Puducherry which includes 12 urban and 15 rural PHCs. Two-stage cluster sampling was adopted to select the participants. In the first stage, simple random sampling was used to select one PHC from rural and one from urban. Subsequently, purposive sampling was used to select the two clusters from each PHC. There were 2228 eligible couples aged between 18–49 years present in these 4 clusters, and all were included in the study.

Initially, Mettupalayam PHC from urban Puducherry and Koodapakkam PHC from rural Puducherry were selected by simple random technique. Then, two areas were selected from the already selected two PHCs which included Shanmugapuram and Sonarpet areas attached to Mettupalayam PHC (Urban) and Koodapakkam and Konerikuppam areas from Koodapakkam PHC (Rural). These were selected purposively and we assume that this selection procedure will result in minimal bias because of homogeneity in socio-demographic and other characteristics in selected PHCs.

### Method of data collection

Baseline data was collected for a period of one year (from 30.08.2016 to 29.08.2017) by a trained Auxiliary Nurse Midwife and supervised by investigators. Sociodemographic characteristics and household details were collected from all the currently married eligible couples using a pre-tested questionnaire. It also captured the women's personal information like include number of living children, number of living sons, age at marriage, urban or rural area, distance to health facility, family type, number of abortions etc. House to house visits were done to reach the participants. If the participants were not able to meet in first house visit, further two visits were made to reach them. Women who were not approachable even after three visits were considered as non-respondents. The questionnaire was prepared based on National Family Health Survey, India18 and revised Uday Parik scale was used to assess the socio-economic status.

Fertility desire was assessed by asking the question “Would you like to have a child or would you prefer not to have any more children?” If somebody says ‘yes’ then they have asked about “How long would you like to wait from now before the birth of another child?” The responses to question are: (a) would like to have a child (b) prefer not to have a child anymore; (c) cannot get pregnant; (d) undecided or don't know. Respondents, who want another child are then asked: “How long would you like to wait from now before the birth of another child?” The answer includes (a) number of months (b)now (c) I cannot get pregnant (d) undecided. The waiting period was categorised as now, 0 to 12 months, and 13 to 24 months which was included for calculating current fertility desire. More than 24 months waiting period was an unmet need for spacing methods. Data regarding other associated factors with current fertility desire which include number of living children, number of living sons, age at marriage, urban or rural area, distance to health facility, family type, number of abortions were collected.

### Statistical analysis

Data were entered in Excel spreadsheet and analyzed by using STATA version 14 (StataCorp. Texas, United States) was used for analysis. The women with the fertility desire were expressed with proportions with 95% Confidence Intervals (CI). Univariate analysis was performed and unadjusted prevalence ratios (UPR) with 95% CI was calculated. Variables with a p-value less than 0.2 in the univariate analysis were included in the generalised linear regression analysis model to estimate the adjusted prevalence ratios (APR). A p-value of less than 0.05 was considered statistically significant.

## Results

Overall, 1979 eligible couples participated with 55 pregnant mothers. Nearly one-third of them (606, 30.6%) were aged between 18–29 years and a quarter of them (569,28.8%) were 40–49 years. Majority aged at marriage by 18 to 24 years of age (66.3%, n=1312) and more than half of them (54.4%, n = 1076) had 2 children. Nearly, 80% (1576) were belonged to low socioeconomic status, and half 51.2%, 1014) were educated more than 10^th^ standard. (Table 1)

**Table 1:** Socio-demographic characteristics of currently married eligible couples in urban and rural area of Puducherry, south India (N=1979)

Associated factors	n	%
**Age (in years)**		
18–24	175	8.8
25–29	431	21.8
30–34	415	21.0
35–39	389	19.7
40–49	569	28.8
**Education of mother**		
No schooling	284	14.4
1st to 10th standard	681	34.4
>10th standard	1014	51.2
**Socio economic status**		
Low	1576	79.6
Moderate	392	19.8
High	11	0.6
**Occupation**		
Housewife	1664	84.1
Others (Skilled/unskilled/professional)	315	15.9
**Religion**		
Hindu	1946	98.3
Muslim	7	0.4
Christian	26	1.3
**Family type**		
Nuclear	1416	71.6
Joint/Extended	563	28.4
**Number of children preferred**		
1	139	7.0
2	1528	77.2
3	286	14.5
≥4	26	1.3
**No of living children**		
0	179	9.0
1	421	21.3
2	1076	54.4
≥3	303	15.3
**Number of living son**		
0	620	31.3
1	931	47.0
≥2	428	21.6
**Only girl child**		
No	1531	77.36
Yes	448	22.6
**H/O Abortion**		
No	1675	84.6
One time	231	11.7
Two times or more	73	3.7
**Age at marriage (in years)**		
18 to 24	1312	66.3
25 and above	667	33.7
**Years of married life**		
1 to 2	184	9.3
3 to 4	134	6.8
≥5	1661	83.9
**Distance to health facility in km**		
<1	1326	67.0
1 to 3	653	33.0
**Area of residence**		
Urban	1126	56.9
Rural	853	43.1

**Figure 2:**
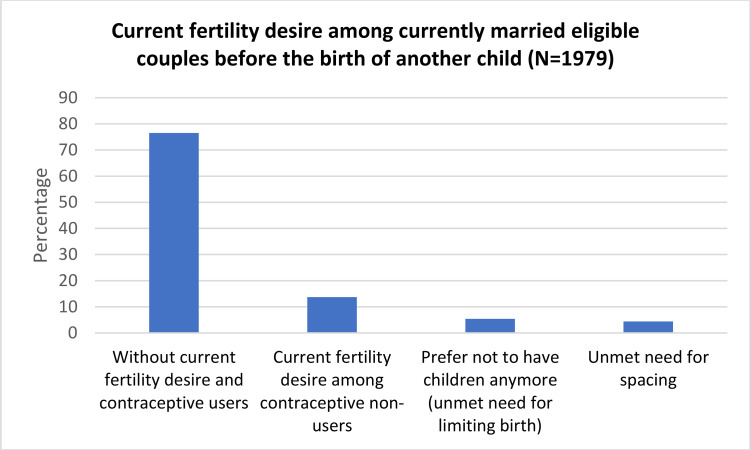
Current fertility desire among currently married eligible couples before the birth of another child

**Figure 3:**
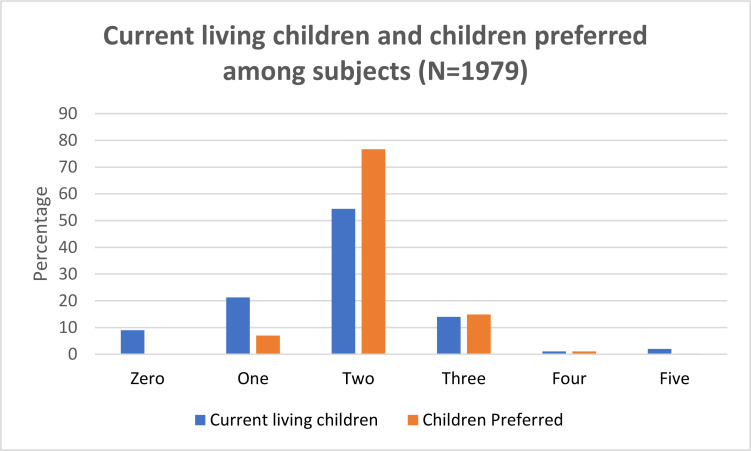
Current living children and children preferred among subjects (N=1979)

Current fertility desire within next two years was found among 13.7% (95% CI -12.3% – 15.3%) of the respondents [Figure 2]. Out of 1979 eligible couples, 1513(76.5%) were currently using contraception methods and don't have current fertility desire, 107 (5.4%) had unmet need for limiting birth and 87 (4.4%) had unmet need for spacing. Mean number of children (SD) currently living and preferred was 1.77(0.851) and 2.11 (0.528) respectively. Majority of them preferred two children (76.7%, 1517) [Figure 3]. Age, education of the women, socio-economic status, religion, family type, number of living children, number of living son, married life, distance thealth facility and residence were significantly associated with fertility desire in univariate analysis [Table 2].

**Table 2:** Associated factors of fertility desire among eligible couples (N=1979)

Associated factors	Number of eligible couples	Number of subjects with fertility desire (%)	Chi square, P value
Age group (in years) 18–24 25–29 30–34 35–39 40–49	175 431 415 389 569	75(42.9) 105(24.4) 49(11.8) 28(7.2) 15(2.6)	240.693, <0.001*
Education of mother No schooling 1^st^ to 10^th^ standard >10^th^ standard	284 681 1014	20(7.0) 52(7.6) 200(19.7)	62.776, <0.001*
Socio economic status Low Moderate High	1576 392 11	188(11.9) 80(20.4) 4(36.4)	23.812, <0.001*
Occupation Housewife Others(Skilled/unskilled/professional)	1664 315	235(14.1) 37(11.7)	1.262, 0.261
Religion Hindu Muslim Christian	1946 7 26	261(13.4) 1(14.3) 10(38.5)	13.582, 0.001*
Family type Nuclear Joint/Extended	1416 563	156(11.0) 116(20.6)	31.23, <0.001*
Number of living children 0 1 2 ≥3	179 421 1076 303	136(76.0) 122(29.0) 12(1.1) 2(0.7)	855.705, <0.001*
Number of living son 0 1 2 or more than 2	620 931 428	185(29.8) 83(8.9) 4(0.9)	213.02, <0.001*
Only girl child No Yes	1531 448	220(14.4) 52(11.6)	2.292, 0.130
H/O Abortion No One time Two times or more	1675 231 73	227(13.6) 38(16.5) 7(9.6)	2.542, 0.281
Age at marriage (in years) 18 to 24 25 and above	1312 667	168(12.8) 104(15.6)	2.898, 0.089
Married life 1 to 2 years 3 to 4 years 5 years and above	184 134 1661	101(54.9) 47(35.1) 124(7.5)	369.438, <0.001*
Distance to health facility <1km 1 to 3km	1326 653	160(12.1) 112(17.2)	9.544, 0.002*
Area Urban Rural	1126 853	138(12.3) 134(15.7)	4.883, 0.027*

* P value less than 0.05 is considered as statistically significant.

After adjusting for confounders, age of 18–24 (APR= 2.91), 25–29 years (APR=2.48), 30–34 (APR=2.47), 35–39 (APR=2.06), high socioeconomic status (APR=2.02), those without child (APR=52.35) and those with one child (APR=35.60) were significantly associated with fertility desire with the p-value of less than 0.05 [Table 3].

**Table 3:** Associated factors of current fertility desire: Generalised linear regression analysis (N=1979)

Associated factors	Number of eligible couples	Number of subjects with fertility desire n (%)	Unadjusted PR (95% CI)	Adjusted PR (95% CI)	p value
**Age group (in years)**					
18–24	175	75(42.9)	16.26 (9.59–27.56)	2.91 (1.77–4.81)	<0.001
25–29	431	105(24.4)	9.24 (5.25–15.64)	2.48 (1.52–4.03)	<0.001
30–34	415	49(11.8)	4.48 (2.55–7.87)	2.47 (1.51–4.05)	<0.001
35–39	389	28(7.2)	2.73 (1.48–5.04)	2.06 (1.24–3.41)	0.005
40–49	569	15(2.6)	Reference	Reference	-
**Education of mother**					
No schooling	284	20(7.0)	Reference	Reference	-
1st to 10th standard	681	52(7.6)	1.08 (0.66–1.78)	0.90 (0.61–1.33)	0.608
>10th standard	1014	200(19.7)	2.80 (1.80–4.35)	0.93 (0.66–1.32)	0.694
**Socio economic status**					
Low	1576	188(11.9)	Reference	Reference	-
Moderate	392	80(20.4)	1.71 (1.34–2.16)	0.96 (0.80–1.16)	0.701
High	11	4(36.4)	3.05 (1.37–6.74)	2.02 (1.24–3.29)	0.004
**Occupation**					
Housewife	1664	235(14.1)	1.20 (0.87–1.66)	-	-
Others	315	37(11.7)	Reference	-	-
**Religion**					
Hindu	1946	261(13.4)	Reference	Reference	-
Muslim	7	1(14.3)	1.07 (0.17–6.56)	1.23 (0.87–1.75)	0.238
Christian	26	10(38.5)	2.87 (1.74–4.72)	1.25 (0.89–1.76)	0.189
**Family type**					
Nuclear	1416	156(11.0)	Reference	Reference	-
Joint/Extended	563	116(20.6)	1.87 (1.50–2.33)	1.09 (0.91–1.30)	0.351
**Number of living children**					
0	179	136(76.0)	115.1 (28.85–459.27)	52.35 (13.08–209.49)	<0.001
1	421	122(29.0)	43.90 (10.94–176.16)	35.60 (8.17–155.0)	<0.001
2	1076	12(1.1)	1.69 (0.38–7.51)	1.38 (0.32–6.02)	0.666
≥3	303	2(0.7)	Reference	Reference	-
**Number of living son**					
0	620	185(29.8)	31.92 (11.95–85.29)	1.66 (0.55–4.97)	0.364
1	931	83(8.9)	9.54 (3.52–25.84)	0.98 (0.32–2.96)	0.975
2 or more than 2	428	4(0.9)	Reference	Reference	-
**Only girl child**					
No	1531	220(14.4)	2.28 (1.74–2.98)	0.56 (0.27–1.17)	0.128
Yes	448	52(11.6)	Reference	Reference	-
**H/O Abortion**					
No	1675	227(13.6)	1.41 (0.69–2.89)	-	-
One time	231	38(16.5)	1.71 (0.80–3.67)	-	-
Two times or more	73	7(9.6)	Reference	-	
**Age at marriage (in years)**					
18 to 24	1312	168(12.8)	Reference	-	-
25 and above	667	104(15.6)	1.22 (0.97–1.53)	-	-
**Married life**					
1 to 2 years	184	101(54.9)	7.35 (5.93–9.11)	0.92 (0.73–1.15)	0.463
3 to 4 years	134	47(35.1)	4.69 (3.53–6.25)	1.03 (0.79–1.35)	0.816
5 years and above	1661	124(7.5)	Reference	Reference	
**Distance to health facility**					
<1km	1326	160(12.1)	Reference	Reference	-
1 to 3km	653	112(17.2)	1.42 (1.14–1.77)	1.10 (0.91–1.33)	0.314
**Area**					
Urban	1126	138(12.3)	Reference	Reference	-
Rural	853	134(15.7)	1.28 (1.02–1.60)	1.16 (0.96–1.40)	0.132

*P value less than 0.05 is considered as statistically significant.

## Discussion

This research study among currently married eligible couples found that 13.7% of them currently desired to have another child. The younger age group, without or with one child and high socioeconomic status group had more fertility desire. It was observed that majority of eligible couples are lesser than 25 years age and completed two-child norm. Most of them had their preferred number of children. The study gives information on the extent of need for fertility services required according to the felt needs of the eligible couples. The findings will be useful for concerned stakeholders to understand what proportion of eligible couples requires fertility-related services within next two years period and need for unmet need for spacing methods for those who had fertility desire after two years period. Other studies showed higher fertility desire than this study 20, 22,23. About 57.1% of the mothers had fertility desire in Uganda study where subjects were recruited during couples' HIV counselling 23. Most studies were conducted among HIV mothers showed majority had fertility desire 12–17. Sub Saharan Africa study conducted in Ghana, Ethiopia, and Nigeria which assessed fertility desire in future life showed that majority of the couples had fertility desire 20. All the above studies assessed fertility desire in future life of eligible couples. A study conducted in Kenya, Nigeria and Senegal found that 13.6%, 18.2% and 17.8% of eligible couples wanted the child within two years, which is similar to this study 19. The lesser fertility desire in this study may be because it assessed the fertility desire within next two years period of interview. Besides majority had completed their desired family size and use contraceptive devices.

Kenya, Senegal, and Nigeria study also found that among all women in all three countries, they wanted to have child but also wants to delay a pregnancy two or more years 19. A study in Nairobi slum area showed that large majority of women who wanted child in future (66.7%), wanted to delay the next child and very few wanted a child soon (2%), one-fifth of the women wanted to wait for two to four years, and more than one-third wanted to wait for at least five years. A small fraction of the women was undecided about future childbearing^25^. But here, it is comparatively more than this study for those who wants child within two years. Approximately 46% of mothers reported bearing more children as shown from Bangladesh study 26. Recent National Family Health Survey-4 (2015–16) in India found that about 24% of currently married women want to have another child in their future life. About 12% of women want to have a child within two years which is similar to this study 21.

Fertility desire also may be influenced by behavioral factors for postponement of pregnancies. If personal or family conditions remain unfavorable, desires to postpone a birth persist and may lead to long birth intervals. In contrast, short birth intervals may be seen if conditions become favorable sooner than anticipated 27,28.

Australian study among 18 to 30 years married females highlighted the factors which include psychosocial predictors of attitude, pressure from others, and perceived self-confidence as predictors of women's intentions to delay childbearing 24. Place of residence, geographic location, religion, wealth index, maternal age and education, partners' education, experiencing child death, and other empowerment-related indicators were significantly associated with unmet fertility desires in Bangladesh study 26.

Exposure to awareness programs on fertility may change fertility desires and waiting time is one of the important factors influencing fertility and prevention of unwanted pregnancies 29. Among never pregnant married women aged 15–24, 21.49% reported a preferred waiting time for their first childbirth of 2 years or more 30. Evidence-based information on fertility desie and spacing and its application will be useful for improving maternal and child health.

The study found that sex composition of living children on fertility desire was not associated with fertility desire. A study from Malawi, Africa in contrast found that high fertility desire was associated with gender preference and sex composition of the living children 10. The finding that number of living children and younger age group was an important associated factor for fertility desire in this study was established by other studies^22^. It is common to observe that if the couples had not attained their desired family size, they had a higher fertility desire compared to those who attained their desired family size 22. Therefore, these study findings have implications for fertility control programs and it advises to improve service care delivery to eligible couples who have not yet attained their desired family size. At the same time, to ensure that those who do not desire any more children do not get unwanted pregnancies by advising appropriate contraception methods.

We found that educated women had more fertility desires similar to Uganda's study. In contrast, other studies showed that higher education is associated with lower fertility desire 31,32. Education was shown to have wide range of behaviours with most of it has depressing impact on fertility desire, while it can also increase fertility level 32. This may be because of the fact that fertility desire is also influenced by other factors like socio-economic development, social structure, cultural context and society's stage in fertility transition 32. We included socioeconomic status in the regression model to reduce the collinearity between these variables. Further qualitative research may explore the influence of social or cultural factors, which was not assessed in this study.

Larger sample size, rural and urban area inclusion, community-based setting, and fairly representative population were strengths of the study. There are some limitations. Since our study included all the eligible couples from the selected family and cluster, design effect may play a role. Larger sample size and considering homogeneity across the clusters, we expect minimal bias related to design effect. Nonrespondents characteristics also influence the study findings. We could not collect data on social and cultural factors associated with fertility desire. A temporal relationship may be difficult to ascertain because of cross-sectional study. Larger follow up studies may further provide a change in behaviour of fertility desire among eligible couples.

## Conclusion

The fertility desire is comparatively lesser than other studies. Younger age group couples had higher desire compared to older age groups. Those without or with a single child and higher socio-economic status group had more fertility desire. Services should be given to these target groups for their felt need of fertility desire. These findings also suggest the need for strengthening reproductive health care services according to the felt need of these target groups in the population. This study recommends concerned health authority to target these women to provide fertility related services within next two years period and unmet need for spacing services for those who had fertility desire after two years period. Further follow up studies will explore the fertility desire dymamics among the women over a period of time.
